# Minimal amount of exercise prevents incident dementia in cognitively normal older adults with osteoarthritis: a retrospective longitudinal follow-up study

**DOI:** 10.1038/s41598-023-42737-3

**Published:** 2023-10-03

**Authors:** Yu-Hsuan Chung, Cheng-Yu Wei, Ray-Chang Tzeng, Pai-Yi Chiu

**Affiliations:** 1 Department of Orthopedics, Show Chwan Memorial Hospital, Changhua, Taiwan; 2 Department of Exercise and Health Promotion, College of Kinesiology and Health, Chinese Culture University, Taipei, Taiwan; 3 Department of Neurology, Chang Bing Show Chwan Memorial Hospital, Changhua, Taiwan; 4 Department of Neurology, Tainan Municipal Hospital (Managed By Show Chwan Medical Care Corporation), Tainan, Taiwan; 5 Department of Neurology, Show Chwan Memorial Hospital, 542, Sec 1, Chung-Shan Rd., Changhua, 500 Taiwan; 6 Department of Applied Mathematics, Tunghai University, Taichung, Taiwan

**Keywords:** Medical research, Neurology

## Abstract

Robust evidence suggests that regular exercise, including walking more than 6000 steps, is effective for preventing dementia; however, such activity is less feasible in older people with osteoarthritis (OA) or other motor disabilities. Therefore, we aimed to test whether the minimal amount of exercise (MAE) could help prevent dementia in older adults with OA. A retrospective longitudinal study was performed and a non-demented cohort (≥ 50-years-old) of 242 people (155 [64.0%] non-converters and 87 [36.0%] converters) from three centers in Taiwan was analyzed with a mean follow-up of 3.1 (range 0.3–5.9) and 2.9 (range 0.5–6.0) years, respectively. MAE was defined as walking for approximately 15–30 min or 1500–3000 steps. Rate of MAE (0, 1–2, or ≥ 3) within one week were defined as MAE-no, MAE-weekly, or MAE-daily, respectively. The incidence rates of dementia were compared between groups. Multivariate logistic regression and Cox proportional hazards analyses were used to study the influence of MAE on dementia occurrence. Age, education, sex, activities of daily living, neuropsychiatric symptoms, cognition, multiple vascular risk factors, and related medications were adjusted. Compared to the MAE-no group, the odds ratios for the incidents of dementia were 0.48 and 0.19 in the MAE-weekly and MAE-daily groups, respectively. In addition, older age, poorer cognition, poorer ADL performance, and congestive heart failure increased the incidence of dementia. Daily and weekly MAE prevented dementia in older adults with OA. As such, an informative public health policy may help promote adequate exercise in at-risk groups.

## Introduction

Robust evidence suggests that regular aerobic exercise has preventative effects on not only on cardiovascular diseases (CVD), but also on degenerative brain disorders—including senile dementia^1–4^. Several recent studies on large populations in the community have provided evidence that even regular walking of up to 4000 steps helps prevent dementia and mortality^5–7^. Therefore, regular exercise, especially aerobic exercise, has been suggested in multiple clinical criteria for the prevention or treatment of disorders^8^ and in public health policies for health promotion^9^. However, there exist barriers to exercise among older adults. In a study focusing on community-dwelling elderly individuals, it was found that health problems and pain were the most frequently reported barriers to exercise^10^. Similarly, the study by Booth et al.^11^ revealed that both poor health and injury were identified as major obstacles to exercise participation among inactive elderly Australians aged 60 to 78 years. Physicians play a crucial and central role in promoting exercise behavior among the elderly^12^. However, there are common barriers that hinder physician intervention, including limited time during office visits, inadequate reimbursement for preventive counseling, and insufficient training and perceived effectiveness in behavioral counseling^13^. Furthermore, one of the major barriers to realizing the potential benefits of long-term exercise adoption among adults with knee osteoarthritis (OA) is the generally low adherence^14^. Pain, body mass index, and mental health which are important factors associated with disabling knee OA, have been found to correlate with exercise adherence^15^.

Dementia is the most common degenerative brain disorder affecting approximately 10% of the elderly population worldwide. Dementia greatly impacts patients and caregivers, making the prevention of the disorder a public health priority. Motor dysfunction due to different etiologies is also commonly observed in older individuals, and the resultant pain or disability may decrease motivation which ultimately causes worsening of pain and disabilities. The minimal amount of exercise (MAE) refers to the smallest amount of physical activity that can provide health benefits or reduce mortality^16^. MAE and exercise can be distinguished according to the following rules^17^: Exercise is often performed with a specific purpose, such as improving cardiovascular fitness. MAE may lack a clear purpose and may not target specific fitness goals. Exercise typically involves moderate to high intensity, which means it raises heart rate and breathing rate. MAE may not. Furthermore, exercise is usually performed for a sustained period, typically at least 30 min, to achieve health benefits. MAE may be brief and sporadic. Finally, exercise is generally performed regularly, multiple times per week. MAE may occur infrequently or irregularly. In this study, the reported minimum exercise level was 15 min a day or 90 min a week^16^.

Several studies have found that regular physical activity positively affects cognitive function in older adults. For example, a 2018 systematic review and meta-analysis found that physical activity, including low-intensity activities, such as walking, was associated with improved cognitive function in older adults. The present study also found that a higher level of physical activity was associated with a lower risk of cognitive impairment and dementia^18^. Another study published in 2019 found that even light-intensity physical activity, such as household chores or leisurely walking, was associated with a lower risk of cognitive decline in older women^19^. Among various physical activities, walking instilled confidence in everyone as it was a familiar and straightforward activity they had been engaging in throughout their lives^20^. The possible mechanism between walking and dementia could be attributed to their impact on overall vitality and biological aging^21^. Additionally, there may be other mechanisms, such as the connection between cardiovascular health and dementia^22^, the correlation with preclinical dementia, and potentially direct influences on brain plasticity as well as the structural and functional reserves of the brain^23,24^.

While the exact amount and type of exercise needed to prevent cognitive decline may vary depending on individual factors, such as age and overall health, evidence suggests that any amount of physical activity can be beneficial^5,6,16^. Therefore, encouraging older adults to engage in regular physical activity, even in minimal amounts, can help improve their cognitive health and quality of life. Few studies have addressed the effects of MAE on the prevention of dementia^25^; and there have been no investigations addressing the impact of MAE in term of walking on the prevention of dementia. Therefore, we aimed to test whether MAE would help prevent dementia among older adults with OA.

## Methods

### Participants

This was a retrospective, longitudinal, follow-up cohort study. Data from the History-Based Artificial Intelligence Clinical Dementia Diagnostic System (HAICDDS) project were used^26,27^. In the data base, consecutive series of older adults who at least 50-years-old were recruited from three centers in Taiwan.

#### Assessment tools

1. Dementia was diagnosed according to the criteria for dementia proposed by the National Institute on Aging and Alzheimer’s Association (NIA-AA)^28^. Participants with impairments in two or more cognitive domains and a decline in daily functions according to the Clinical Dementia Rating (CDR) scale, and the Cognitive Abilities Screening Instrument (CASI).
2. The CDR scale was used to diagnose the cognitive impairment severity, including dementia. Participants with a global CDR score ≥ 0.5 plus a CDR-SB score ≥ 2.5 and with impairments in two or more cognitive domains in the CDR plus a decline in daily functions are considered to have dementia^29–31^.
3. The Cognitive Abilities Screening Instrument (CASI) was used to assess cognitive performance. It comprises 9 cognitive domains, has been validated and widely utilized in Taiwan, as well as many other countries, with the use of various languages^32^. The cut-off score should be in range for dementia after adjusting for age, sex, and education^32^.
4. The History-Based Artificial Intelligence Activities of Daily Living scale (HAI-ADL), which is composed of 15 questions and can be applied in individuals with both instrumental and basic ADL impairments due to cognitive disorders for the determination of severity of CI stages^33^.
5. Definition of MAE: The minimal dose for reducing dementia was 3826 steps^5^. As previously mentioned, the study reported a minimum exercise level of either 15 min per day or 90 min per week^16^. According to these prior research findings, MAE was defined as approximately 15–30 min of walking or 1500–3000 steps. MAE scores of 0, 1–2, or ≥ 3 within one week were defined as MAE-no, MAE-weekly, or MAE-daily, respectively.
6. Other neuropsychological assessments including the Montreal Cognitive Assessment (MoCA)^34^ and the Neuropsychiatric Inventory (NPI)^35^ were used for comparison among different groups. Each of these assessments has undergone validation and reliability testing.

### Procedures

**Figure 1:**
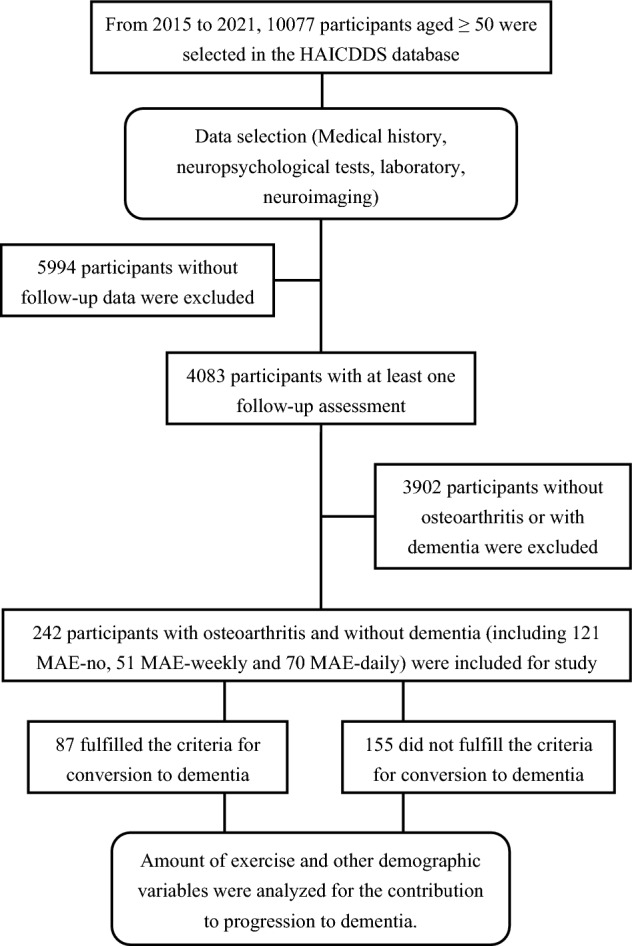
Flow chart for selecting the study participants.

The incidence rates of dementia among different MAE were compared. Multivariate logistic regression and Cox proportional hazards analyses were used to study the influence of MAE on the incidence of dementia. Age, education, sex, activities of daily living, neuropsychiatric symptoms, cognition, multiple vascular risk factors, and related medications were adjusted for. All participants were recruited from 2015 to 2021. Based on the purpose of the registration of the HAICDDS database, a consecutive series of participants registered to the database were included for further criteria for inclusion or exclusion. Among the participants in the HAICDDS database, 10077 participants aged ≥ 50 were initially selected. After a thorough data selection, which included medical history, neuropsychological tests, laboratory results, and neuroimaging scans, 5994 participants without follow-up data were excluded. The remaining 4083 participants with at least one follow-up assessment were included. Those without a history of osteoarthritis or had been diagnosed with dementia were also excluded. Finally, 242 participants with osteoarthritis and without dementia were included in the study, comprising 121, 51 and 70 in the MAE-no, the MAE-weekly and the MAE-daily groups, respectively. The detailed inclusion and exclusion criteria, along with the participant characteristics, are summarized in Fig. 1.

### Data analysis

The Chinese version of SPSS (version 22.0; IBM Corp., Armonk, NY, USA) was used for the statistical analyses. The background characteristics of different MAE groups were compared. The comparisons included cognition according to the CASI^32^. The Montreal Cognitive Assessment (MoCA)^34^, History-Based Artificial Intelligence Activities of Daily Living scale (HAIADL)^33^, Neuropsychiatric Inventory (NPI)^35^, clinical history of hypertension, hyperlipidemia, coronary artery disease (CAD), arrhythmia, congestive heart failure (CHF), cerebrovascular disease (CVD), atherosclerosis, smoking, and exercise, were compared. Current medications, including antihypertensives, lipid-lowering drugs (LLD), anti-diabetes, antiplatelets, and anticoagulants, were also compared.

Multiple logistic regression analysis was used to investigate the contribution of the conversion from normal cognition to dementia according to MAE frequency. The MAE-no group was set as the reference group, and odds ratios (OR) were adjusted for age, sex, education, follow-up years, CASI, HAIADL, NPI, diabetes, hypertension, dyslipidemia, coronary artery diseases, arrhythmia, congestive heart failure, and medications. Cox proportional hazards regression was used to investigate the contribution of the frequency of MAE among non-dementia participants to conversion to dementia. HR were compared with those in the MAE-No group and adjusted for age, sex, education, CASI, HAIADL, NPI, diabetes, hypertension, dyslipidemia, coronary artery disease, arrhythmia, congestive heart failure, and all medications. All calculated *p*-values were two-tailed. Statistical significance was defined as a *p*-value < 0.05.

### Ethical consideration

This cohort study was conducted retrospectively, and the selected data were processed and analyzed anonymously. The Institutional Review Board of Show Chwan Memorial Hospital approved this study and waived the informed consent requirement (SCMH_IRB No: IRB1081006). Research has been performed in accordance with the Declaration of Helsinki and Good Clinical Practice.

## Results

A total of 242 participants were analyzed, including 155 (64.0%) non-converters and 87 (36.0%) converters with a mean follow-up of 3.1 (range 0.3–5.9) and 2.9 (range 0.5–6.0) years, respectively. Among them, 121, 51, and 70 patients experienced no, weekly, and daily MAE, respectively. In this population, 50% of the participants had at least MAE-weekly. The comparison of demographic variables among the three groups without adjustment revealed significant differences in the follow-up years (*p* = 0.011) and the other variables were not significant (Table 1).

**Table 1:** Comparison of the background characteristics among the different frequencies of minimal amount of exercise (MAE).

MAE Group	Daily (≥ 3 per week)	Weekly (1–2 per week)	No (< 1 per week)	*F*/χ^*2*^	*p*
*n*	70	51	121		
Age, year, mean (SD)	73.7 (8.9)	75.1 (8.4)	74.2 (9.6)	0.31	0.71
Female, *n* (%)	37 (52.9)	34 (66.7)	55 (54.5)	2.71	0.26
Education, year (SD)	5.1 (4.9)	4.2 (4.2)	5.5 (4.3)	1.40	0.25
CDR-SB	1.8 (1.3)	2.0 (1.3)	2.2 (1.4)	2.03	0.13
CASI, mean (SD)	71.9 (15.4)	68.0 (14.9)	67.9 (18.4)	1.39	0.25
MoCA, mean (SD)	16.3 (6.7)	14.1 (6.7)	14.1 (6.9)	2.59	0.08
HAIADL, mean (SD)	3.9 (2.9)	4.2 (2.7)	4.9 (3.4)	2.21	0.11
NPI, mean (SD)	3.3 (5.5)	5.0 (6.3)	4.7 (6.1)	1.66	0.19
Follow-up year, mean (SD)	2.7 (1.6)	3.1 (1.5)	2.3 (1.4)	4.59	0.011
Clinical history, *n* (%)					
Hypertension	52 (74.3)	41 (80.4)	89 (73.6)	0.95	0.62
Diabetes	24 (34.3)	16 (31.4)	54 (44.6)	3.52	0.17
Dyslipidemia	37 (52.9)	19 (37.3)	45 (45.5)	2.91	0.23
CAD	10 (14.3)	7 (13.7)	12 (9.9)	0.99	0.61
Arrythmia	11 (15.7)	14 (27.5)	20 (16.5)	3.37	0.19
CHF	7 (10.0)	7 (13.7)	16 (13.2)	0.77	0.77
Atherosclerosis	29 (41.4)	21 (41.2)	48 (39.7)	0.07	0.97
Social activities	27 (38.6)	19 (37.3)	53 (43.8)	0.86	0.65
Current medication, *n* (%)					
Antihypertensives	54 (77.1)	41 (80.4)	91 (95.2)	0.55	0.76
Anti-diabetes	16 (22.9)	14 (27.5)	40 (33.1)	2.31	0.32
LLDs	35 (50.0)	23 (45.1)	6 (49.6)	0.35	0.84
Anti-depressants	14 (20.6)	12 (23.6)	22 (18.2)	0.66	0.72
Hypnotics	25 (36.8)	26 (51.0)	54 (44.6)	2.47	0.13
Analgesics	29 (42.6)	24 (47.1)	56 (46.3)	0.30	0.86

*n* number of cases, *ns* non-significance, *CDR-SB* sum of boxes of the Clinical Dementia Rating scale, *CASI* Cognitive Abilities Screening Instrument, *MoCA* Montreal Cognitive Assessment, *HAIADL* History-Based Artificial Intelligence Activities of Daily Living scale, *CAD* coronary artery disease, *CHF* congestive heart failure, *LLDs* lipid-lowering drugs.

**Figure 2:**
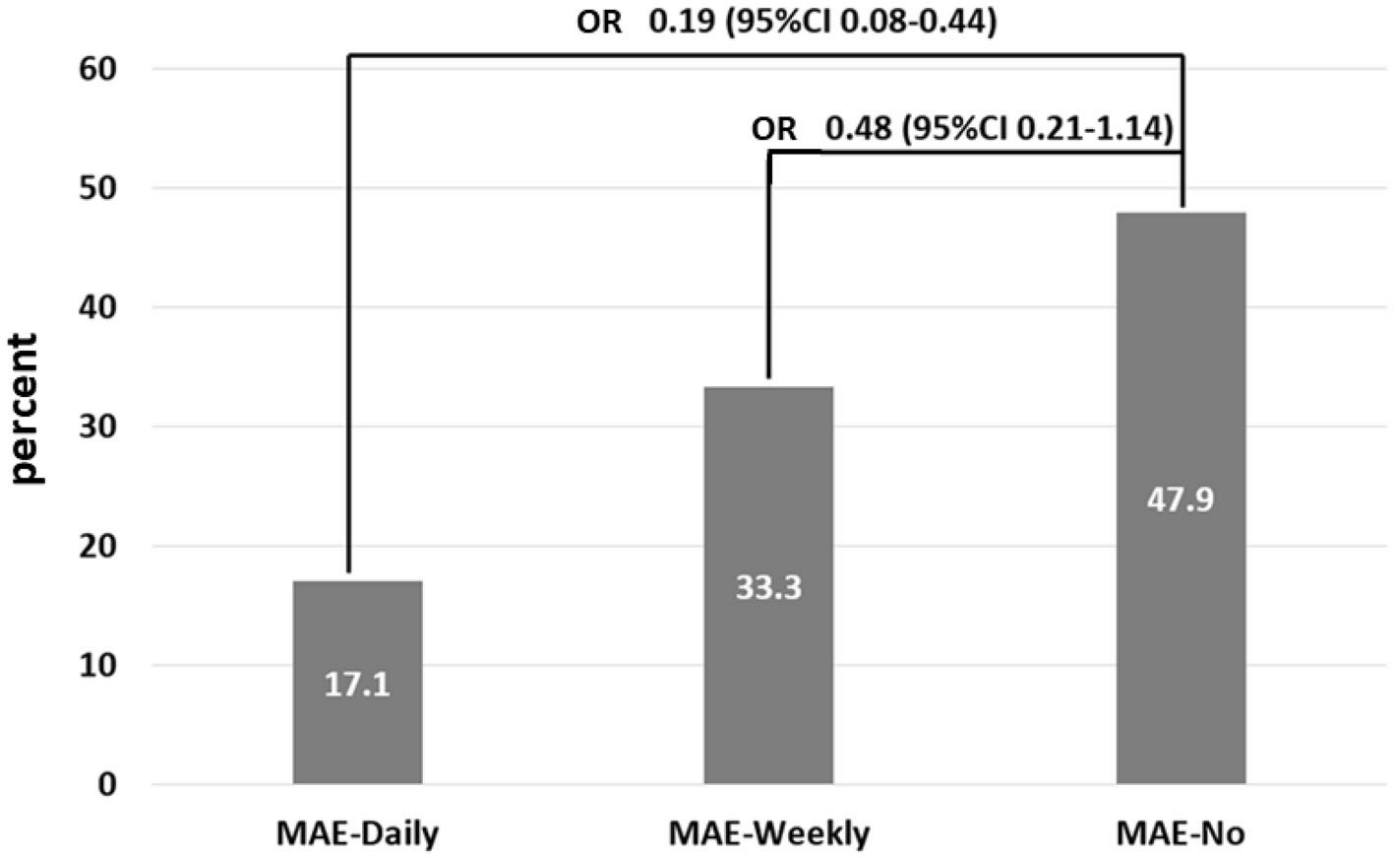
Percentage frequency of conversion according to the MAE frequency. Multiple logistic regression analysis was adopted to investigate the contribution of frequency in the non-dementia participants to conversion to dementia. OR were adjusted for age, sex, education, follow-up years, CASI, HAIADL, NPI, diabetes, hypertension, dyslipidemia, coronary artery diseases, arrythmia, congestive heart failure, and medications. Compared to MAE-No group, OR were 0.48 and 0.19 in the MAE-weekly group and MAE-daily group, respectively.

The percentage conversion rates were 47.9%, 33.3%, and 17.1% for the MAE-no, MAE-weekly, and MAE-daily groups, respectively. Figure 2 shows the percentage frequency of conversion according to the MAE frequency. Multiple logistic regression analysis was used to investigate the contribution of the frequency of non-dementia participants to conversion to dementia. OR were adjusted for age, sex, education, follow-up years, CASI, HAIADL, NPI, diabetes, hypertension, dyslipidemia, coronary artery disease, arrhythmia, congestive heart failure, and medications. Compared to people in MAE-no group after adjustment, the OR for incident dementia were 0.48 (95% CI = 0.21–1.14; *p* = 0.096) and 0.19 (95% CI = 0.08–0.44; *p* < 0.001) in those with MAE-weekly and MAE-daily, respectively (Fig. 2). In addition, female sex decreased incident dementia (OR = 0.42; 95% CI = 0.19–0.88; *p* = 0.022). However, poorer ADL performance increased incident dementia (OR = 1.18; 95% CI = 1.03–1.35; *p* = 0.014).

**Figure 3:**
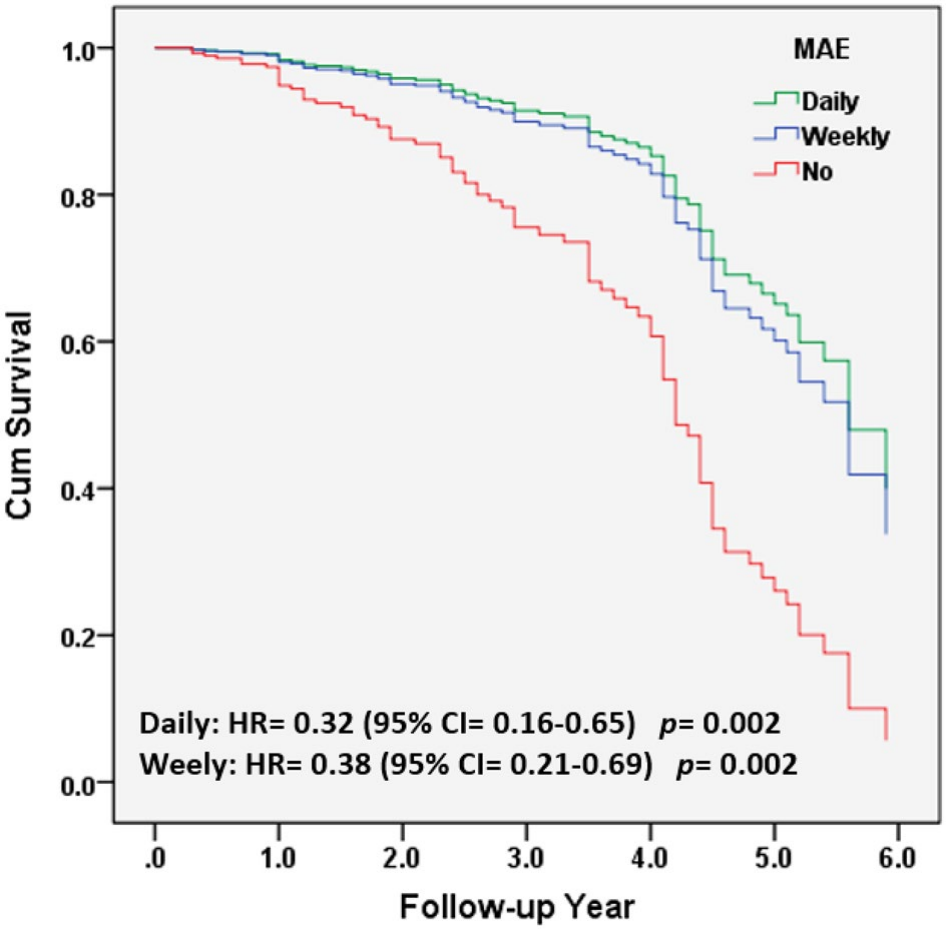
Cox proportional hazards regression was adopted for investigating the contribution of the MAE frequency among non-dementia participants to the dementia conversion group. HR were compared with MAE-No group and were adjusted for age, sex, education, CASI, HAIADL, NPI, diabetes, hypertension, dyslipidemia, coronary artery diseases, arrythmia, congestive heart failure, and all medications.

Figure 3 demonstrates that Cox proportional hazards regression was adopted to investigate the contribution of the frequency of MAE among non-dementia participants to conversion to dementia. The HR were compared with those of the MAE-No group and adjusted for age, sex, education, CASI, HAIADL, NPI, diabetes, hypertension, dyslipidemia, coronary artery diseases, arrhythmia, congestive heart failure, and all medications. Compared to people in MAE-no group after adjustment, the HR for incident dementia were 0.38 (95% CI =  0.21–0.69; *p* = 0.002) and 0.32 (95% CI = 0.16–0.65; *p* = 0.002) in those with MAE-weekly and MAE-daily, respectively (Fig. 3).

Finally, Cox proportional hazards regression was adopted to investigate the contribution of MAE and other variables of participants without dementia to the conversion to dementia. HR was compared with that of the non-converter group after adjusting for age, sex, education, cognition (CASI), activities of daily living (HAIADL), neuropsychiatric symptoms (NPI), diabetes, hypertension, dyslipidemia, coronary artery disease, arrhythmia, congestive heart failure, and all medications (Table 2). In addition, male sex and poor ADL performance increase the incidence of dementia.

**Table 2:** Cox proportional hazards regression was adopted for investigating the contribution of MAE and other variables in the no-dementia participants to the dementia-converted-patients.

Variables	B	Wald	Sig	Exp	95% CI for Exp
MAE					
No	0				
Weekly	− 0.97	9.88	0.002	0.38	0.21–0.69
Daily	− 1.14	9.98	0.002	0.32	0.16–0.65
Age	0.03	2.46	0.117	1.03	0.99–1.06
Sex	− 0.76	5.61	0.018	0.47	0.25–0.88
Education	0.06	2.90	0.088	1.07	0.99–1.14
CASI	− 0.03	7.30	0.007	0.97	0.96–0.99
HAIADL	0.11	6.34	0.012	1.11	1.02–1.21
NPI	0.01	0.09	0.768	1.01	0.97–1.04
Diabetes	− 0.51	1.69	0.193	0.60	0.28–1.29
Hypertension	− 0.44	0.79	0.375	0.64	0.24–1.71
Dyslipidemia	− 0.32	1.06	0.303	0.73	0.40–1.33
coronary artery diseases	0.21	0.33	0.567	1.24	0.60–2.58
Arrythmia	0.16	0.22	0.640	1.18	0.60–2.31
congestive heart failure	0.90	6.19	0.013	2.45	1.21–4.95
Antihypertensives	− 0.34	0.43	0.512	0.72	0.26–1.95
Anti-diabetes	7.13	2.76	0.097	2.04	0.88–4.74
Lipid-lowering drugs	− 0.12	0.19	0.666	0.89	0.51–1.54
Anti-depressants	0.12	0.16	0.687	1.29	0.63–2.04
Hypnotics	− 0.48	3.23	0.073	0.62	0.37–1.05
Analgesics	0.25	0.92	0.337	1.13	0.79–2.16

HR were compared with MAE-No group and were adjusted for age, sex, education, CASI, HAIADL, NPI, diabetes, hypertension, dyslipidemia, and coronary artery diseases, arrythmia, congestive heart failure, and all medication.

*CASI* Cognitive Abilities Screening Instrument, *HAIADL* History-based Artificial Intelligence Activities of Daily Living, *NPI* Neuropsychiatric Inventory. *B* beta, *Wald* Wald test, *Sig* significance or *p* value, *Exp* exponential, *95% CI* 95% confidence interval.

## Discussion

OA is a common condition in older adults and is associated with a higher risk of cognitive decline and dementia^36,37^. However, regular physical activity has been shown to have a positive impact on both joint health and cognitive function in older adults with OA^38,39^. OA is a condition that causes joint pain and stiffness and can limit a person’s mobility and ability to engage in physical activity. Regular exercise can help maintain joint function and improve overall health, including cognitive health. Studies have shown that physical exercise can improve cognitive function in older adults with OA^38,39^. To investigate the influence and frequency of MAE on the prevention of incident dementia, this study provided several important findings. The conversion rates to dementia were 47.9%, 33.3%, and 17.1% for the MAE-no, MAE-weekly, and MAE-daily groups, respectively. After adjusting for age, education, sex, cognitive and ADL functions, health condition, and medication, both the MAE-daily and MAE-weekly groups showed significant reductions in the incidences of dementia compared to the MAE-no group using the Cox proportional hazards model. Additionally, using multivariate logistic regression, MAE-daily significantly reduced incidences of dementia compared to the MAE-no group. These findings are consistent with those of previous studies, suggesting that even minimal amounts of exercise can help prevent cognitive decline in elderly people with OA^40–42^. For example, a study published in 2017 found that a home-based exercise program improved the cognitive function of older adults with knee OA^40^. The program included low-intensity exercises, such as stretching, balance exercises, and low-impact aerobics, which can be performed even in individuals with mobility limitations. Therefore, encouraging elderly people with OA to engage in regular physical activity, even in minimal amounts, can help improve their joint and cognitive health.

In addition to exercise in older people with OA, this study demonstrated that older age, poor cognition, poor ADL performance, and congestive heart failure significantly increased the incidence of dementia after adjusting for all related factors in the Cox proportional hazards model. These findings are consistent with those of several previous studies. Old age is a well-established risk factor for dementia. The risk of developing dementia increases with age, and the prevalence of dementia doubles approximately every five years after 65 years of age. Several studies have examined the relationship between age and incident dementia and have consistently found that older age is a strong predictor of dementia^43–45^. 5.3% of individuals aged 65 to 74, 13.8% of individuals aged 75 to 84, and 34.6% of individuals aged 85 and older are affected by Alzheimer's dementia^43^. De Reuck et al.^46^ demonstrated that the primary age of the mixed Alzheimer's disease (AD) group was significantly higher compared to the pure AD group, with mean ages of 77 years (SD = 10) and 69 years (SD = 10), respectively (*p* ≤ 0.001). Previous studies have suggested that poor cognition is associated with an increased risk of incident dementia^19,47^. Evidence suggests that congestive heart failure may increase the risk of incident dementia as well^48,49^. Dementia presents a growing public health concern, underscoring the urgency to identify interventions that can effectively prevent or delay the onset of dementia for older adults with OA. This study provides valuable insights that can influence interventions designed to support older adults with dementia and OA in incorporating ‘walking’ into their daily routines. This low-volume of physical activity could play a pivotal role in the implications for public health policies globally fighting against dementia and leading to decreased healthcare expenses and addressing health inequalities.

In conclusion, this study highlights that both daily and weekly MAE have a significant impact on reducing the incidence of dementia in older adults with OA. This finding was the first to our knowledge to shed light on the benefits of regular walking for older adults with OA. These results emphasize the importance of implementing a comprehensive public health policy aimed at preventing physical and mental decline, particularly among older adults with varying motor functions or abilities. Although growing evidence links exercise and demographics or vascular risk factors to incident dementia, the exact mechanisms by which these risk factors contribute to cognitive decline and dementia have not yet been fully elucidated. Further research is required to identify the underlying pathways and mechanisms that link vascular risk factors to dementia, which may lead to the development of more effective preventive and treatment strategies.

This study has some limitations. First, the study population was relatively small and the study was conducted at only three centers in Taiwan. Therefore, a selection bias should be considered. Second, the comparison of the MAE association with incident dementia was a longitudinal follow-up, but due to the design being retrospective instead of prospective, the causal relationship has not been clarified sufficiently. Third, estimated  sample size was not calculated before starting the study. Finally, the number of covariates should be carefully considered for having robust results; thus, sensitivity analysis may be needed in the future to verify the robust of the results. Further prospective longitudinal follow-ups in a larger cohort recruited from more centers with sample size calculation before the study are warranted.

## Data Availability

All data and material are included in the manuscript.
